# Investigation into the efficiency and prognostic elements of CT-guided ¹²⁵I particle implantation for liver cancer

**DOI:** 10.1186/s40644-025-00909-6

**Published:** 2025-07-11

**Authors:** Yuxiao Xia, Quanyu Zhou, Xue Jiang, Wenling Tu, Qian Liu, Liangshan Li, Yuhong Shi

**Affiliations:** 1 Department of Nuclear Medicine, Second Affiliated Hospital of Chengdu Medical College (China National Nuclear Corporation 416 Hospital), Chengdu, 610000 Sichuan P.R. China; 2 Department of Hepatobiliary and Pancreatic Surgery, Chengdu 363 Hospital Affiliated to Southwest Medical University, Chengdu, 610041 Sichuan P.R. China; 3 Department of Minimally Invasive Interventional Medicine, First Affiliated Hospital of The Army Medical University, Chonqing, 400030 P.R. China

**Keywords:** Hepatocellular carcinoma (HCC), Iodine-125 (¹²⁵I) seed implantation, CT-guided therapy, Local progression-free survival (LPFS), Overall survival (OS)

## Abstract

**Purpose:**

This study assessed the effectiveness and prognostic factors of CT-guided ¹²⁵I seed implantation for primary hepatocellular carcinoma (HCC).

**Methods:**

A retrospective analysis of 71 patients (57 males, 14 females, median age 64) treated at three Chinese hospitals from 2018 to 2024 was conducted. The main outcomes were local progression-free survival (LPFS) and overall survival (OS). Treatment involved a 16-slice Spiral CT and Radioactive Particle Treatment Planning System (TPS), with seeds of 18.5–29.6 MBq implanted via freehand puncture. Efficacy was evaluated using the modified Response Evaluation Criteria in Solid Tumors (mRECIST) at three months, with follow-ups every three months for three years, then biannually until December 2024. Data analysis utilized SPSS 22.0, Kaplan-Meier, and Cox models.

**Results:**

With a median follow-up of 37 months, the complete response (CR) rate was 57.7%, partial response (PR) 31.0%, stable disease (SD) 5.6%, and progressive disease (PD) 5.6%. Local control was 94.3%. LPFS rates at 1, 3, and 5 years were 74.6%, 29.5%, and 1.4% (median LPFS 22 months), while overall survival (OS) rates were 88.7%, 47.8%, and 12.6% (median OS 35 months). CR was a key protective factor for LPFS and OS. Significant factors included the Barcelona Clinic Liver Cancer (BCLC) stage C, intrahepatic progression, and extrahepatic metastasis. Postoperative complications occurred in 35.2% of patients, with no severe cases.

**Conclusion:**

CT-guided ¹²⁵I seed implantation is effective and safe for primary HCC, with CR being crucial for survival. Large-scale studies are needed to confirm these results.

## Introduction

Hepatocellular carcinoma (HCC) represents a significant contributor to cancer-related mortality on a global scale, characterized by consistently high incidence and mortality rates. According to global cancer statistics liver cancer ranks as the sixth most prevalent cancer worldwide and the third leading cause of cancer-related deaths, with an estimateld 866,136 new cases and 758,725 fatalities annually [1, 2]. Reports indicate that the five-year survival rate among patients with HCC is alarmingly low, falling below 15% [3]. At present, the therapeutic modalities for high-risk hepatocellular carcinoma (HCC) encompass surgery, local ablation, transarterial chemoembolization (TACE), chemotherapy, immunotherapy, and molecular targeted therapy, yet each of these approaches harbors inherent limitations [4]. Although improved physical examination levels have increased the chances of early detection and subsequent radical surgery for HCC patients, radical resection is only applicable to 10-37% of liver cancer cases, as most patients are diagnosed at an advanced stage [5]. Moreover, the recurrence rate within 5 years after radical resection is as high as 70% [6]. Radiofrequency ablation (RFA) is one of the commonly used radical treatment methods for small HCCs, and its clinical efficacy is comparable to that of radical resection. However, for lesions with a maximum diameter of ≥ 5 cm and those located in high-risk anatomical areas, the risk of residual lesions after ablation treatment increases, thereby affecting overall survival (OS) [7]. Transarterial chemoembolization (TACE) is an important treatment option for intermediate and advanced liver cancer patients, but it faces issues such as incomplete embolization, tumor microenvironment hypoxia, and acidification, which increase the risk of postoperative recurrence and metastasis and also affect the efficacy of subsequent immunotherapy. The complete remission rate for patients receiving TACE alone is less than 2% [8]. Chemotherapy, immunotherapy, and targeted therapy have been proven to significantly improve the progression-free survival (PFS) and overall survival (OS) of advanced HCC patients, but drug side effects, drug resistance, and high costs remain significant challenges [9]. Therefore, there is an urgent need to develop more effective therapeutic strategies to improve the prognosis of patients with HCC.

In recent years, iodine-125 (^125^I) seed implantation therapy, has emerged as a prominent form of localized radiotherapy for HCC, attracting considerable attention within the medical community. Iodine-125 (¹²⁵I) particles represent a form of low-radiation element preparation characterized by a half-life ranging from approximately 59.5 to 60.14 days, during which they emit low-energy γ-rays continuously. When these particles are implanted directly into tumor tissues, the emitted γ-rays precisely target cancer cells, disrupting the double-stranded structure of their DNA. This disruption effectively inhibits mitosis, curtails uncontrolled cellular proliferation, and induces apoptosis. This targeted radiotherapeutic strategy optimizes the eradication of tumor cells while minimizing damage to adjacent healthy tissues [10, 11]. Clinical research has demonstrated that CT-guided implantation of iodine-125 particles is both highly effective and safe for the treatment of patients with high-risk hepatocellular carcinoma (HCC) [12, 13]. Furthermore, the combination of iodine-125 particle implantation with transarterial chemoembolization (TACE) has been shown to yield synergistic effects, significantly prolonging the overall survival (OS) and progression-free survival (PFS) of patients with HCC [14]. Compared with radiofrequency ablation (RFA) alone, the use of iodine-125 particle implantation following RFA demonstrates superior efficacy in treating hepatic tumor lesions [15]. The use of ^125^I seed implantation therapy has demonstrated favorable efficacy and safety profiles in various other types of tumors, including small cell lung cancer, pulmonary metastatic tumors, head and neck soft tissue sarcomas, retroperitoneal liposarcomas, cervical cancer, malignant parotid gland tumors and maxillary sinus carcinoma [16–25].

Harnessing precise targeting and high-dose localized radiation, ^125^I seed implantation therapy offers a minimally invasive, safe, and highly effective treatment modality for patients. This study aims to evaluate the clinical efficacy and prognostic factors of ^125^I seed implantation therapy for primary hepatocellular carcinoma (HCC) by assessing local progression-free survival (LPFS) and overall survival (OS). We anticipate that the findings of this study will provide robust scientific evidence and valuable guidance for clinical treatment strategies.

## Subjects and methods

### General information

A total of 71 patients with hepatocellular carcinoma who underwent CT-guided ^125^I seed implantation in three hospitals in China (The Second Affiliated Hospital of Chengdu Medical College, Chengdu 363 Hospital Affiliated to Southwest Medical University, The First Affiliated Hospital of Army Medical University) from June 2018 to June 2024 were retrospectively enrolled. The cohort comprised 57 males and 14 females, with a median age of 64 years (range: 34–78 years). Among these patients, 41 (57.7%) presented with a single intrahepatic lesion, 16 (22.5%) had two lesions, and 14 (19.8%) had three lesions. The median alpha-fetoprotein (AFP) level was 16.45 µg/L (interquartile range: 3.45, 136.67 µg/L), and the median maximum tumor diameter was 3.9 cm (interquartile range: 2.8, 6.8 cm). In our study, the cohort of 71 patients was stratified into two distinct groups: the initial treatment group and the residual or recurrent group. The initial treatment group consisted of 28 patients (39.4%), each presenting with high-risk liver cancer deemed unsuitable for surgical resection or ablation, or who had declined transarterial chemoembolization (TACE). The residual or recurrent group included 43 patients (60.6%). It is noteworthy that this classification is specific to the criteria employed in our study and may not align with more recent classifications documented in the literature. The criteria for inclusion and exclusion of patients with hepatocellular carcinoma in this study are detailed in Table 1 [26].

**Table 1:** Inclusion and exclusion criteria for patients

Inclusion Criteria	Exclusion criteria
a) Hepatocellular carcinoma diagnosed clinically or histopathologically. b) Candidates unsuitable for or declining surgery. c) Lesions in the diaphragm dome, near the gallbladder, or gastrointestinal tract are ineligible for ablation. d) Residual or recurrent tumors post-RFA or TACE are included. e) Child-Pugh Class A or B. f) Up to three lesions, with a single lesion < 7 cm or multiple lesions (< 5 cm each, max three). g) Barcelona Clinic Liver Cancer Stage A-C.	a) More than three lesions. b) Prior ^125^I seed implantation for intrahepatic lesions. c) Expected survival < 6 months. d) Uncorrectable coagulopathy. e) Severe combined organ dysfunction.

### Equipments and materials for ^125^I particles implantation therapy

#### The equipment and instruments

The study employed a 16-slice Spiral CT and the Pinpoint Piercing Guidance System, both manufactured by Philips in the Netherlands. Additionally, a Radioactive Particle Computer Treatment Planning System (TPS) was utilized, sourced from Beijing University of Aeronautics and Astronautics. The ^125^I radioactive particles, model 6711-eob99, were supplied by Beijing Yatom Hi-Tech Co., Ltd. These particles are specified by their dimensions of 4.5 mm in length and 0.8 mm in diameter, a half-life of 59.6 days, and an average photon energy between 27 and 35 keV. The prescribed dose for these particles ranges from 90 to 160 Gy, with a tissue penetration distance of 1.7 cm and a particle activity between 18.5 and 29.6 MBq.

#### Process and status of particle implantation

An enhanced upper abdominal computed tomography scan with 5 mm slices was performed within three days prior to surgery, and the resulting data were uploaded to the Radioactive Particle Computer Treatment Planning System. The gross tumor volume (GTV) and organs at risk (OAR) were delineated in accordance with the guidelines outlined in “Abdominal Tumor Radioactive Particle Therapy Techniques”. The determination of iodine-125 seed activity prior to implantation should be informed by a thorough assessment of factors such as target volume, proximity to organs at risk (OARs), tumor differentiation grade, and treatment objectives. Personalized adjustments should be made to optimize therapeutic efficacy while minimizing harm to normal tissues. For tumors with a diameter of less than 5 cm that are situated near critical structures (e.g., hepatic hilar vessels, bile ducts, gastrointestinal tract) or are well-differentiated, the seed activity is generally set between 18.5 and 22.2 MBq. In contrast, for tumors exceeding 5 cm in diameter, located at a distance from critical organs, or poorly differentiated, the seed activity is typically set between 25.9 and 29.6 MBq. The trajectory of the needle, including its position, angle, and depth, was meticulously planned, and particle distribution was simulated to ensure a minimum spacing of 1 cm and dose supplementation at adjacent levels. Patients were appropriately positioned and immobilized, and liver lesions were scanned using CT slices of 0.5 cm thickness. The implantation of ^125^I particles was executed via freehand puncture, guided by either a grid or laser system. Puncture points and trajectories were preoperatively planned in the TPS, followed by disinfection and administration of local anesthesia. An 18-gauge particle needle was inserted at 1 cm intervals, and particles were implanted in a “retrograde” manner at 0.5–1.0 cm intervals in accordance with the TPS. A final computed tomography scan was performed to evaluate particle distribution, with reseeding conducted as necessary. Postoperative imaging was analyzed within the TPS for dosimetric evaluation, comparing it with preoperative parameters, specifically focusing on D_90_ and D_100_, which represent the dose received by 90% and 100% of the GTV, respectively. Grid localization was used in 47 cases (66%), and laser localization was used in 24 cases (34%). The particle activity was 18.5 MBq in 3 cases (4%), 22.2 MBq in 15 cases (21%), 25.9 MBq in 41 cases (58%), and 29.6 MBq in 12 cases (17%). A total of 36 (21, 77) seeds were implanted, with postoperative D_90_ of 128.6 (121.4, 147.9) Gy and postoperative D_100_ of 71.3 (52.6, 83.1) Gy.

#### Efficacy evaluation

Short-term efficacy was assessed three months post-surgery using the modified Response Evaluation Criteria in Solid Tumors (mRECIST). Complete response (CR) was defined as the disappearance of all target lesions for at least four weeks; partial response (PR) was characterized by a reduction of more than 30% in the sum of the longest diameters of baseline lesions for at least four weeks; stable disease (SD) indicated a decrease in the sum of the longest diameters of baseline lesions that did not meet the criteria for PR, or an increase that did not meet the criteria for progressive disease (PD); PD was defined as a 20% increase in the total length of baseline lesions or the appearance of new lesions. The disease control rate (DCR) was calculated as the sum of CR, PR, and SD. Follow-up was conducted through outpatient visits or telephone interviews, with assessments every three months during the first three years post-implantation, and every six months from the fourth to the sixth year, continuing until December 2024. Local Progression-Free Survival (LPFS) was defined as the duration from the date of seed implantation to either intrahepatic progression or death from any cause, or the date of the last follow-up for patients who were lost to follow-up. Overall Survival (OS) was similarly defined as the interval from the date of seed implantation to death from any cause or the last follow-up for patients lost to follow-up. All adverse events were documented and classified in accordance with the Common Terminology Criteria for Adverse Events, as established by the National Institutes of Health in the United States [27].

### Statistical analysis

Data analysis was performed using SPSS version 22.0. Quantitative variables that were not normally distributed, including follow-up and survival times, were presented as medians with interquartile ranges (Q1, Q3). Qualitative data were expressed as frequencies and percentages. Univariate survival analysis was conducted using the log-rank test, while multivariate analysis was performed using the Cox proportional hazards model. For subgroup analysis between two groups, the Student’s t-test was utilized, whereas analysis of variance (ANOVA) was employed for comparisons involving three or more subgroups. Survival curves were generated using the Kaplan-Meier method, and PFS and OS rates were subsequently calculated. All *P*-values were two-tailed, with statistical significance defined as *P* < 0.05.

## Results

### Analysis of clinical efficacy

As of December 2024, follow-up was conducted for 37 (17, 52) months with a follow-up rate of 100%. There were 33 survivors (46.5%) and 38 deaths (53.5%). The complete response (CR) rate was 57.7% (41/71), the partial response (PR) rate was 31.0% (22/71), the stable disease (SD) rate was 5.6% (4/71), and the progressive disease (PD) rate was 5.6% (4/71); the local control rate was 94.3% (67/71). The patient’s LPFS rates at 1, 3, and 5 years were 74.6%, 29.5%, and 1.4%, respectively, with a median LPFS duration of 22 (12, 38) months. The overall survival (OS) rates at the same intervals were 88.7%, 47.8%, and 12.6%, with a median OS of 35(23, 48) months. Among the 28 patients who received initial treatment, the median LPFS duration was 25 (16, not reached) months, and the median OS was 47 (26, not reached) months, with OS rates at 1, 3, and 5 years reported as 94.1%, 59.6%, and 27.1%, respectively. In contrast, the cohort of 43 patients with residual or recurrent disease exhibited a median LPFS duration of 13 (7, 23) months and a median OS of 15 (9, 49) months, with OS rates at 1, 3, and 5 years of 85.2%, 40.1%, and 3.2%, respectively. Out of 52 patients who experienced disease progression after particle implantation therapy, 55.7% (29/52) had intrahepatic recurrence or metastasis, and 44.2% (23/52) had extrahepatic metastasis or combined intrahepatic recurrence and extrahepatic metastasis. Following progression, 55.7% (29/52) received local treatment (TACE, ablation, or particle implantation therapy), 15.3% (8/52) received systemic therapy (targeted therapy alone, immunotherapy, or in combination with local treatment), and 29.0% (15/52) received palliative supportive care.

### Postoperative complications and causes of death

35.2% (25/71) of patients experienced postoperative complications, including 20 cases of hepatic hemorrhage, all of which improved with symptomatic treatment; 4 cases of pleural effusion, 6 cases of pneumothorax, 2 cases of ascites, and one case each of sepsis, liver abscess, and obstructive jaundice, all of which improved after treatment. There were no cases of liver failure, hepatic rupture, or enteric fistula. A total of 53.5% (38/71) of patients died, with potential causes of death including local progression (10 cases), distant metastasis (12 cases), and others due to diabetic nephropathy, cerebral hemorrhage, infection, trauma, and pulmonary embolism.

### Analysis of LPFS and OS in patients with tumors and their associated factors

The univariate analysis data on LPFS and OS in 71 patients with hepatocellular carcinoma following ^125^I particle implantation are shown in Table 2. Cox univariate analysis of LPFS suggests that the Child-Pugh classification of liver function, the presence or absence of extrahepatic metastasis, the presence or absence of portal vein tumor thrombus, the maximum diameter of the lesions, the Barcelona Clinic Liver Cancer (BCLC) staging, previous treatment history, and recent therapeutic response are all significantly associated with LPFS (all *P* < 0.05). Then, the significant variables from univariate analysis results were included in multivariate Cox analysis. The results indicated that CR (HR = 0.002, 95% CI: 0.001 to 0.146) is a protective factor for LPFS.

**Table 2:** Impact of univariate analysis on local Progression-Free survival (LPFS) and overall survival (OS)

Characteristics	*n*	LPFS		OS	
		HR(95%CI)	*P*	HR(95%CI)	*P*
**Child Pugh score(A vs. B)**	(48 vs. 23)	0.049(0.017–0.144)	<0.05	0.353(0.172–0.722)	<0.05
**Extrahepatic metastasis (No vs. Yes)**	(47 vs. 24)	0.078(0.027–0.224)	<0.05	0.381(0.183–0.796)	<0.05
**Portal vein (No vs. Yes)**	(58 vs. 13)	0.133(0.056–0.319)	<0.05	0.162(0.072–0.366)	<0.05
**The tumor size(≤4 cm vs. >4 cm)**	(32 vs. 39)	0.085(0.033–0.224)	<0.05	0.401(0.199–0.809)	<0.05
**BCLC stage(A + B vs. C)**	(45 vs. 26)	0.167(0.077–0.360)	<0.05	0.293(0.147–0.585)	<0.05
**Previous Treatment History(Initial treatment vs. Recurrent or residual)**	(28 vs. 43)	0.025(0.005–0.117)	<0.05	0.365(0.181–0.733)	<0.05
**mRECIST ( PR + SD + PD vs. CR)**	(30 vs. 41)	49.577(6.209-395.888)	<0.05	2.588(1.237–5.413)	<0.05
**D 90(≤ 160 Gy vs. >160 Gy)**	(58 vs. 13)	2.014(0.901–4.502)	0.088	2.452(1.117–5.382)	<0.05
**Progress After Treatment**					
None	19				
intrahepatic metastasis,	29			0.186(0.075–0.458)	<0.05
extrahepatic metastasis	23			0.324(0.134–0.782)	<0.05
**Post-progress Treatment**					
local treatment	29			0.019(0.004–0.092)	<0.05
systemic therapy	8			0.038(0.008–0.170)	<0.05
palliative supportive care	15			0.053(0.009–0.302)	<0.05

**Fig. 1:**
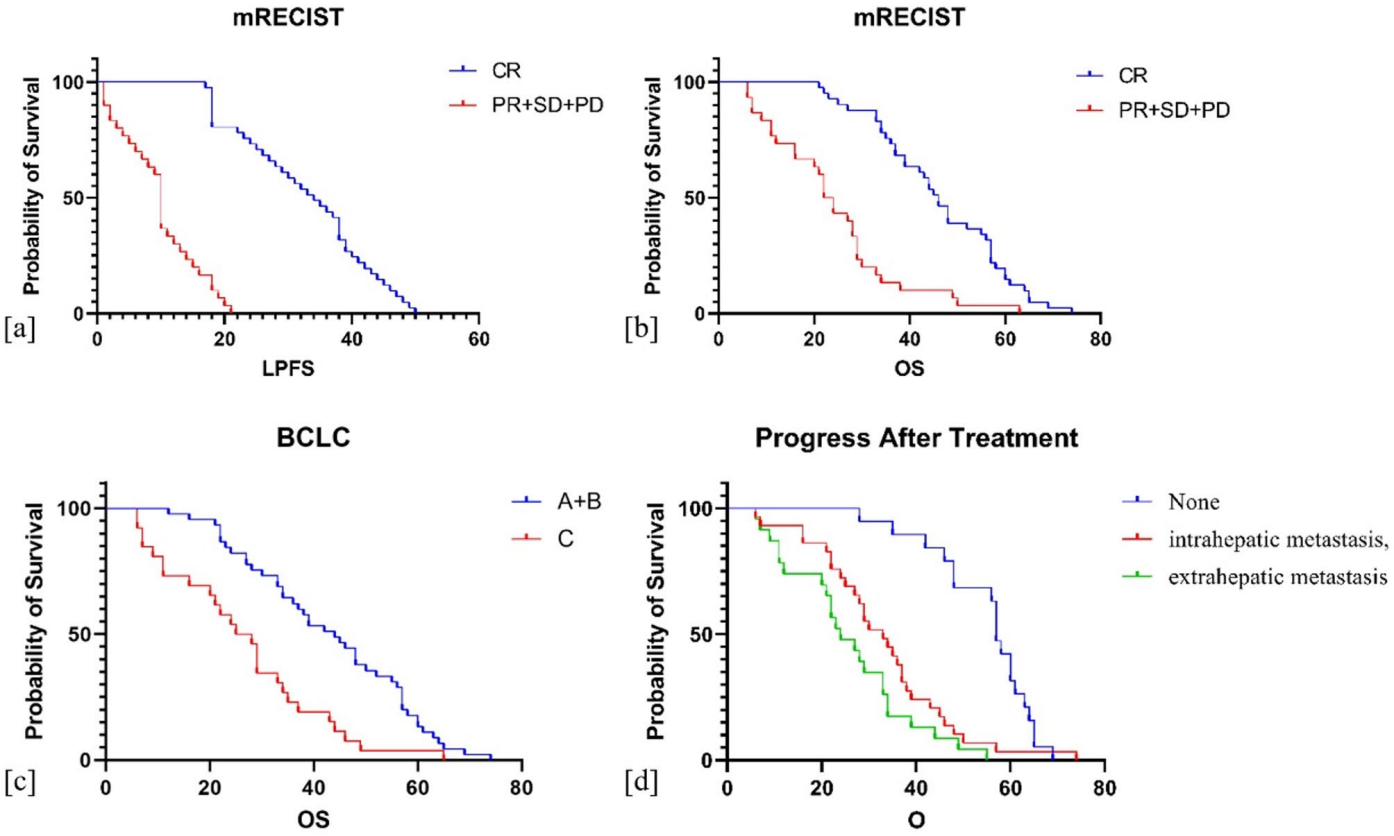
Kaplan-Meier survival analysis in 71 HCC patients post-125I particle implantation therapy. (**a**) LPFS comparison between CR (41 cases) and non-CR groups (30 cases, PR + SD + PD). (**b**) OS comparison between CR (41 cases) and non-CR groups (30 cases). (**c**) OS comparison between BCLC Stage A + B (45 cases) and Stage C (26 cases). (**d**) OS comparison among patients with no progression (19 cases), intrahepatic progression (29 cases), and extrahepatic metastasis (23 cases) following 125I particle implantation

**Fig. 2:**
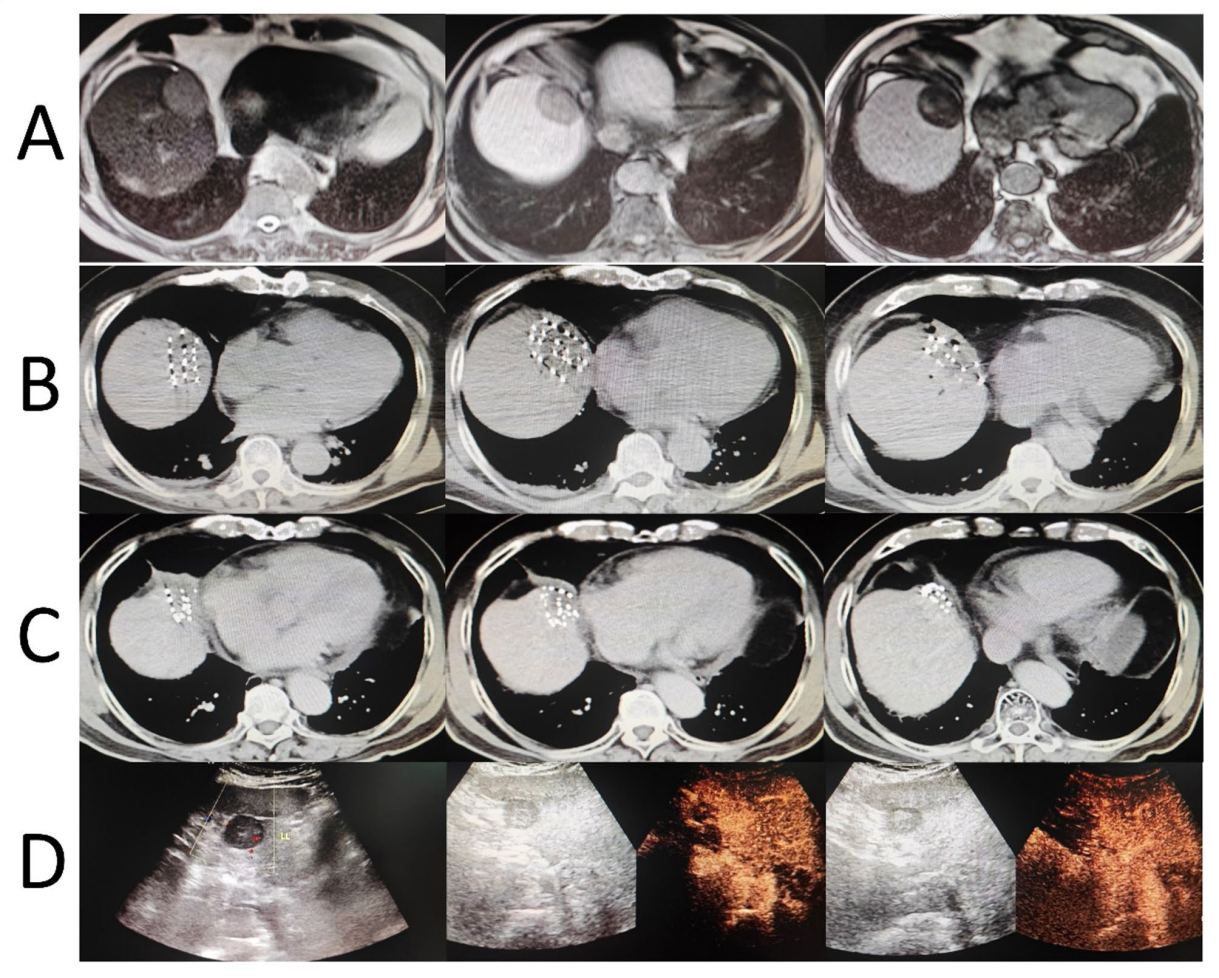
Case of a 72-year-old male patient. In January 2023, he underwent MRI (**A**) due to elevated alpha-fetoprotein (AFP) levels, which revealed a new hepatic lesion in segment VIII (3.6 cm) with restricted diffusion and heterogeneous enhancement. Biopsy confirmed hepatocellular carcinoma. Given the patient’s advanced age and the location of the lesion at the diaphragmatic dome, he underwent CT-guided ^125^I particle implantation for liver cancer in February 2023 (**B**). A follow-up CT scan six months postoperatively (**C**) showed significant reduction in the lesion. In May 2024, an acoustic contrast imaging of abdominal organs (**D**) detected a new hypoechoic lesion in the left liver (2.8 cm), suggestive of liver cancer recurrence. The patient subsequently underwent ultrasound-guided radiofrequency ablation of the hepatic tumor in May 2024. After ^125^I particle implantation, the patient achieved partial remission at three months, with a local progression-free survival (LPFS) of 15 months and an overall survival (OS) of 22 months

**Fig. 3:**
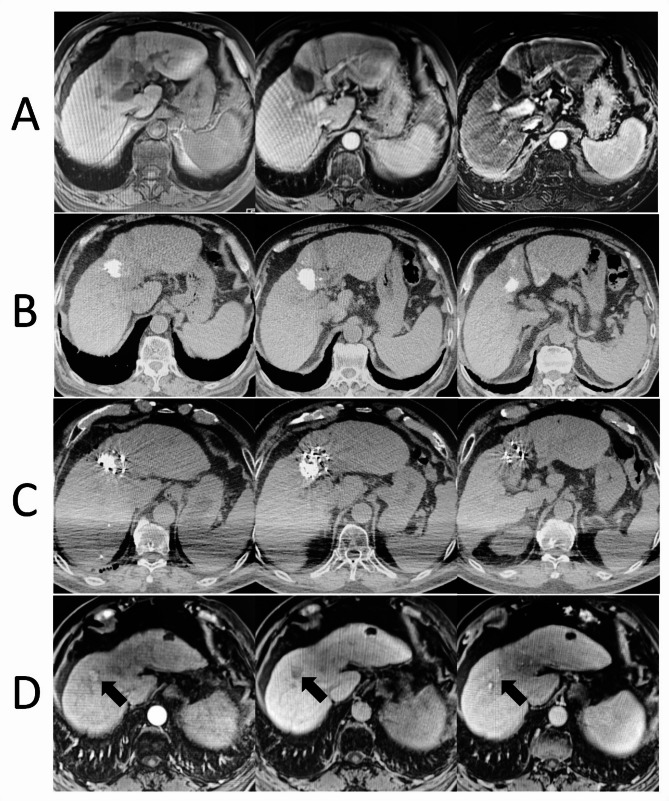
A 71-year-old male patient. In January 2023, a CT scan performed for pneumonia revealed a hepatic segment IV mass (4.2 × 3.3 cm). Biopsy confirmed hepatocellular carcinoma. In February 2023, the patient underwent transcatheter arterial chemoembolization (TACE) and radiofrequency ablation (RFA), followed by oral lenvatinib for anti-tumor therapy. MRI and CT scans in October 2023 showed post-intervention changes (**A** and **B**). However, a significant increase in serum AFP levels suggested local tumor recurrence. In November 2023, based on the patient’s strong preference, a CT-guided ^125^I particle implantation was performed (**C**). Three months post-implantation, the tumor size remained unchanged, but serum AFP levels significantly decreased. In November 2024, MRI revealed a new nodule in hepatic segment VIII, indicative of intrahepatic metastasis (**D**, black arrow). The patient achieved partial response three months after ^125^I particle implantation, with a local progression-free survival (LPFS) of 12 months and an overall survival (OS) of 13 months

Univariate Cox analysis of OS revealed that the aforementioned eight factors, as well as disease progression after particle implantation therapy and subsequent treatment methods, are all significantly associated with OS (all *P* < 0.05). Multivariate analysis results revealed that CR (HR = 0.121, 95% CI: 0.023 to 0.642) is a predictive factor for improved OS, while BCLC stage C (HR = 0.190, 95% CI: 0.061 to 0.595), intrahepatic progression after particle therapy (HR = 0.309, 95% CI: 0.093 to 1.030), and extrahepatic metastasis (HR = 0.042, 95% CI: 0.003 to 0.577) are risk factors, with survival curves shown in Fig. 1. Specific case examples are presented in Figs. 2 and 3.

A subgroup analysis was conducted on 52 patients who progressed after treatment. Among them, the median OS was 33 months for the 29 patients with intrahepatic progression and 24 months for the 23 patients with extrahepatic metastasis (*t* = 1.819, *P* = 0.075). The median OS times for the 29 patients who received local therapy, the 8 patients who received systemic therapy, and the 15 patients who received palliative supportive care were 34, 28, and 16 months, respectively (*F* = 15.684, *P* = 0.016).

## Discussion

In this study, we followed patients with hepatocellular carcinoma who received ¹²⁵I particle implantation therapy and found 1-year, 3-year, and 5-year survival rates of 88.7%, 47.8%, and 12.6%, respectively. These rates were higher than those reported by J. Lv et al., who observed 1-year, 2-year, and 3-year survival rates of 75%, 45.8%, and 27.1% [28]. The difference may be due to our optimized treatment protocol or patient selection. Lin et al. reported even higher 1-year and 2-year survival rates of 95.7% and 82.7% using MRI-guided implantation [29], likely due to MRI’s superior accuracy over CT in assessing liver lesions. In a study on HCC patients with complex tumor locations, the median OS was 33 months, PFS was 30 months, and LCR was 83.33%. Our study showed a median OS of 35 months and a notably higher LCR of 94.3%, underscoring the effectiveness of our treatment [30]. In this study, patients with residual or recurrent lesions after ablation or TACE had a median local progression-free survival (LPFS) of 13 months, significantly longer than the 4.7 to 10.9 months for TACE alone [31]. Other studies show that combining TACE with ¹²⁵I particle implantation increased the complete response rate from 33.3 to 60.5% and extended median overall survival from 17 to 22 months [32]. Adjuvant ¹²⁵I particle implantation post-surgery improved the 5-year survival rate from 29.41 to 55.88% and extended recurrence time from 36.7 to 60 months [29, 33]. This treatment proved effective for local control and survival, offering significant benefits in complex clinical situations and combination therapies, thus serving as a valuable reference for managing HCC.

Recent therapeutic efficacy is a critical determinant of survival following local tumor treatment. Prior research has demonstrated that hepatocellular carcinoma (HCC) patients achieving complete response (CR) have significantly better survival rates [34, 35]. For example, a University of Hong Kong study found a 3-year overall survival (OS) rate of 75.5% for CR patients after locoregional therapy and immunotherapy, compared to 28.1% for non-CR patients [36]. Another study showed a 5-year survival rate of about 50% for CR patients versus 10% for non-CR patients [37]. A report from the Medical University of Vienna presented at the EASL conference noted that although few advanced HCC patients achieved CR with immunotherapy, they had a 5-year OS rate of 77%, with a recurrence rate of 34.5%. This finding is corroborated by our study, in which 58.5% of the patients achieved CR, resulting in a median overall survival (OS) of 46 months and a median local progression-free survival (LPFS) of 34 months. These outcomes were markedly superior to those of patients who did not achieve CR, who had a median OS of 23 months and a median LPFS of 10 months. Furthermore, multivariate analysis identified CR as an independent predictor of enhanced survival outcomes. In patients with HCC, CR serves as a crucial indicator of long-term survival, with rates differing across various treatment modalities. The use of ¹²⁵I particle implantation as a local treatment option enhances the probability of achieving CR, thereby extending patient survival.

The Barcelona Clinic Liver Cancer (BCLC) criteria are the most commonly used staging system for HCC, endorsed by the AASLD and EASL for its thorough assessment of liver function, tumor burden, and prognosis [38]. Numerous studies have validated the BCLC’s prognostic accuracy [39, 40]. This study further confirms that the BCLC system independently predicts overall survival, reinforcing its effectiveness. Hepatocellular carcinoma (HCC) is highly malignant and invasive, often leading to both intrahepatic and extrahepatic metastasis. In this study, 52 patients showed disease progression post-treatment, correlating with poor survival outcomes. The median overall survival (OS) for patients with intrahepatic progression was similar to those with extrahepatic metastasis (t = 1.819, *P* = 0.075), indicating that disease progression adversely affects survival regardless of metastasis type. However, treatment choice significantly impacted survival rates. A propensity score-matched analysis showed that HCC patients undergoing local therapy had significantly better 2-year overall and progression-free survival rates of 66.6% and 47.0%, compared to 31.2% (*p* < 0.001) and 10.6% (*p* = 0.005) for those on systemic therapy [41]. These findings clearly demonstrate that local therapies are superior in controlling disease progression and enhancing patient survival. The study compared patients who received various treatments after disease progression, revealing that those who underwent local therapy had a significantly longer median overall survival compared to those receiving systemic therapy or palliative care (F = 15.684, *P* = 0.016). This underscores the vital role of local therapies in improving survival rates for HCC patients, even after disease progression, highlighting their effectiveness not only in early treatment but also in extending survival thereafter.

Postoperative complications from iodine-125 (¹²⁵I) implantation include bleeding, pain, pneumothorax, leukopenia, radiation-induced liver injury, immune impairment, seed migration, biliary stenosis, enteric and biliary fistulas, and infection [42–44]. In this study, all procedures were successful with no major complications. The most common issue was minor subcapsular hepatic hemorrhage, requiring no intervention. To reduce complications and improve treatment accuracy, careful preoperative planning with imaging techniques is crucial to avoid major vessels and choose the shortest path. For tumors with significant arterial blood supply, preoperative hepatic artery embolization is recommended [45, 46]. Real-time intraoperative monitoring with color Doppler ultrasound significantly reduces complications in high-risk hepatocellular carcinoma (HCC) patients [30].

While this study shows promising results, its limitations must be noted. The retrospective design may introduce selection and information biases, affecting accuracy. A small sample size limits statistical power, possibly hiding the true impact of some prognostic factors. The median follow-up period might not fully reveal long-term efficacy and safety, especially for late recurrence and survival. Additionally, the absence of a standardized treatment protocol across the three hospitals could lead to variability in treatment and outcomes.

## Conclusions

In conclusion, computed tomography (CT)-guided iodine-125 (¹²⁵I) particle implantation for the treatment of hepatocellular carcinoma (HCC) has demonstrated promising short-term efficacy and safety, evidenced by high rates of local control and complete response, which significantly contribute to improved long-term survival outcomes. This study highlights the pivotal role of achieving a complete response in enhancing patient survival. However, due to the retrospective nature and limited sample size of this study, further validation of these findings is necessary. Future research should focus on conducting large-scale prospective studies to comprehensively confirm the efficacy and safety of this therapeutic approach. Moreover, the development of standardized treatment protocols and the optimization of dosimetry are crucial to ensure consistency and reproducibility across diverse clinical settings. Investigating the potential synergistic effects of ¹²⁵I particle implantation in combination with emerging therapies, such as immunotherapy, may provide novel opportunities for optimizing treatment strategies for HCC.

## Data Availability

No datasets were generated or analysed during the current study.
